# Hypoxic preconditioning accelerates the healing of ischemic intestinal injury by activating HIF-1α/PPARα pathway-mediated fatty acid oxidation

**DOI:** 10.1038/s41420-024-01937-0

**Published:** 2024-04-04

**Authors:** Linxia Li, Yanqi Liu, Na Zhi, Yaoxuan Ji, Jialing Xu, Guoyun Mao, Yazhou Wang, Jin Ma, Yunying Wang

**Affiliations:** 1 Department of Aerospace Medicine, Air Force Medical University, 710032 Xi’an, China; 2 Department of Neurobiology and Institute of Neurosciences, Air Force Medical University, 710032 Xi’an, China

**Keywords:** Stem cells, Gastrointestinal diseases

## Abstract

Hypoxic preconditioning (HPC) has been shown to improve organ tolerance to subsequent severe hypoxia or ischemia. However, its impact on intestinal ischemic injury has not been well studied. In this study, we evaluated the effects of HPC on intestinal ischemia in rats. Intestinal rehabilitation, levels of fatty acid oxidation (FAO) by-products, intestinal stem cells (ISCs), levels of hypoxia-inducible factor 1 subunit α (HIF-1α) and its downstream genes such as peroxisome proliferator-activated receptor α (PPARα), and carnitine palmitoyltransferase 1a (CPT1A) were assessed at distinct time intervals following intestinal ischemia with or without the interference of HIF-1α. Our data showed that HPC facilitates the restoration of the intestinal structure and enhances the FAO, by boosting intestinal stem cells. Additionally, HIF-1α, PPARα, and CPT1A mRNA and their protein levels were generally up-regulated in the small intestine of HPC rats as compared to the control group. Our vitro experiment also shows low-oxygen induces highly levels of HIF-1α and its downstream genes, with a concurrent increase in FAO products in IEC-6 cells. Furthermore, the above phenomenon could be reversed by silencing HIF-1α. In conclusion, we hypothesize that HPC can stimulate the activation of intestinal stem cells via HIF-1α/PPARα pathway-mediated FAO, thereby accelerating the healing process post ischemic intestinal injury.

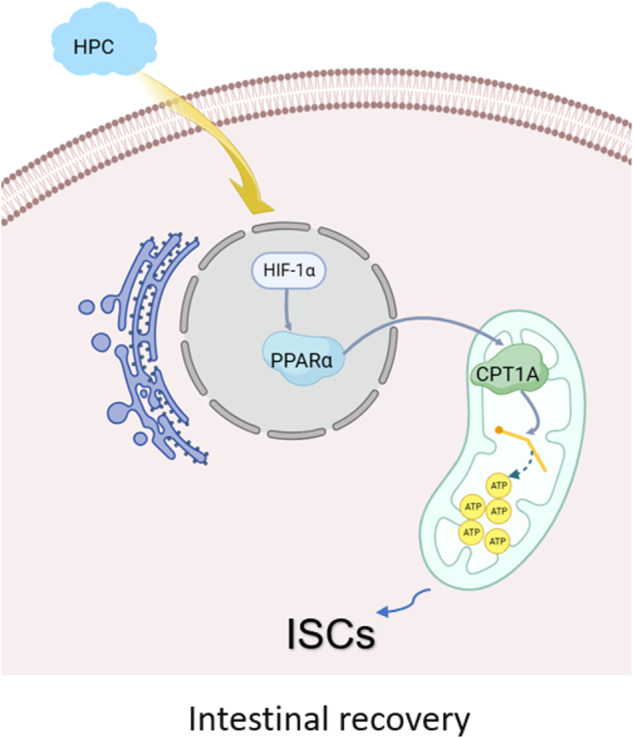

## Introduction

The intestinal mucosa malfunctions due to various factors, such as inflammation, microbial imbalance, severe trauma, hypoxia, and ischemia. Among these, ischemia (IS) is the most common cause in clinical settings. Intestinal ischemia may result in dysfunction of the intestinal mucosal barrier. Failure to alleviate this dysfunction in due time may cause intestinal bacteria to circulate throughout the body, leading to systemic inflammation and multi-organ dysfunction [1, 2].

Intestinal stem cells (ISCs) and Paneth cells collectively establish the intestinal niche, renewing every 3-5 days in order to sustain the epithelial cells. ISCs have the capacity to differentiate into various cell types, thereby replenishing the necessary cells required for the process of renewal [3, 4]. Studies have shown that ISCs rely on energy metabolism and signal transmission. Inhibiting energy production can exert a significant impact on their capacity to carry out vital functions such as organogenesis. Fatty acid oxidation (FAO) plays a pivotal role in situations of ischemic injury, characterized by impaired glucose metabolism, as it promotes the regeneration of ISCs through the activation of specific genes that regulate FAO [5–7]. Certain studies suggest that fasting induces a shift in metabolic pathways towards FAO, resulting in an increase in intestinal stem cell (ISC) production. Conversely, a deficiency in carnitine palmitoyltransferase 1A (CPT1A) affects FAO and leads to a reduction in both the quantity and functionality of ISCs. Fatty acid supplementation also promotes the self-renewal of intestinal organoids. Moreover, it has been observed that the inhibition of this activity adversely affects the function of stem cells [8–13].

Hypoxic preconditioning (HPC) offers significant advantages for individuals. Studies unequivocally demonstrate that the ability to withstand hypoxia and ischemia is significantly enhanced, which is indeed remarkable. HPC plays a crucial role in facilitating the proliferation, differentiation, and migration of mesenchymal stem cells derived from diverse sources [14]. Further evidence suggests that HPC plays a significant role in the repair of repairing bone marrow injuries, facilitating fracture healing, mitigating inflammatory osteolysis, and promoting myocardial recovery after a myocardial infarction. This is achieved through the modulation of extracellular vesicles derived from mesenchymal stem cells, which, in turn, promote angiogenesis [15–17]. The tissue recovery following intracerebral hemorrhage can be improved by enhancing the differentiation capacity of adipose-derived stem cell differentiation capacity through HPC enhancement [18]. The proliferation and differentiation of distinct stem cells are contingent upon the transcriptional modifications induced by HIF-1α and its downstream genes [19, 20]. HIF-1α maintains redox homeostasis equilibrium both at rest and in the presence of oxidative or nutritional stress by persistently stimulating glutaminase-mediated glutathione production. HIF-1α signaling also promotes glycogen synthesis and reduces energy deficits brought on by nutrition or oxygen deprivation [21]. Additionally, angiogenesis and neural axon regeneration following myocardial infarction also benefit from HPC-mediated HIF-1α activation [22, 23].

Although HPC is of great benefit to the body, its role in the repair of intestinal damage has not been reported. Our present primary objective is to gain a comprehensive understanding of the impact of HPC on intestinal injury, along with elucidating the underlying molecular mechanisms responsible for these effects.

## Results

### Promoting the repair of intestinal ischemic injury through HPC

**Fig. 1:**
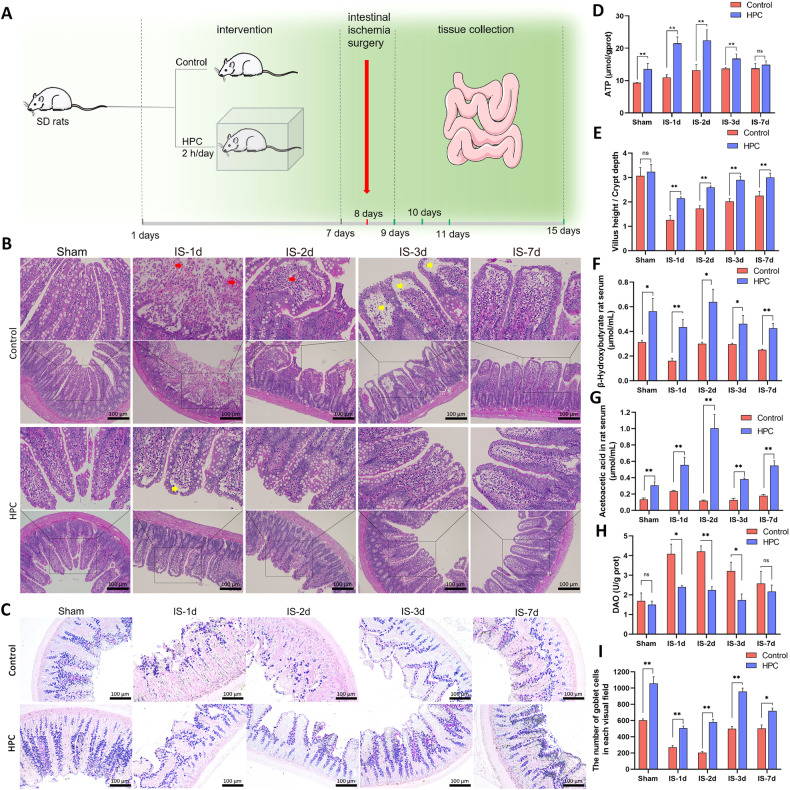
Schematic diagram of animal experiments, H&E images of the intestine, and fatty acid oxidation product contents in rats. **A** Schematic design of the animal experiment. **B**, **E** Ileum H&E staining and the ratio of villus height to crypt depth based on staining in rats. **C**, **I** AB/PAS staining was performed on the rat small intestine to assess goblet cell count. **D** ATP content in small intestine of rats in each group. **F**, **G** β-hydroxybutyrate and acetoacetic acid levels were measured in rats from each group. **H** DAO concentration in serum of each group. Values are listed as the mean ± SD. *p < 0.05, **p < 0.01, and ns, non-significant, compared with the control groups (*n* = 3).

We performed the animal experiments as described in Fig. 1A. Initial villous rupture and hyperemia occur following intestinal ischemia (red arrow). Over time, driven by intestinal stem cells, the integrity of the intestinal villi gradually improves. The formation of the subepithelial space (indicated by the yellow arrow) is a key process in the recovery of intestinal villi. Compared to the control group, the HPC group showed a significant reduction in villi breakage on the day after ischemia, along with a notable decrease in the subepithelial space. By the second day, the villi structure had essentially recovered. In conclusion, HPC treatment significantly accelerated the villi repair process after intestinal ischemia (Fig. 1B, E). The number of goblet cells in the intestine was measured using AB/PAS staining, and the results indicated that the HPC group consistently had a greater number of goblet cells compared to the control group (Fig. 1C, I). We measured the ATP content in the intestinal tissue, and the results showed that HPC significantly increased the ATP content in the intestinal tissue (Fig. 1D). In addition, we also examined the concentrations of β-hydroxybutyrate and acetoacetic acid, which are byproducts of FAO, in the serum of rats. We found that the HPC groups generally had higher concentrations compared to the control groups (Fig. 1F, G). Serum DAO is a highly active intracellular enzyme located in the upper villi of the intestinal mucosa. It reflects the integrity and degree of damage to the intestinal mechanical barrier. The results showed that the concentration of DAO decreased significantly in the HPC group (Fig. 1H).

### Effect of HPC on ISCs in the post-ischemic intestine

**Fig. 2:**
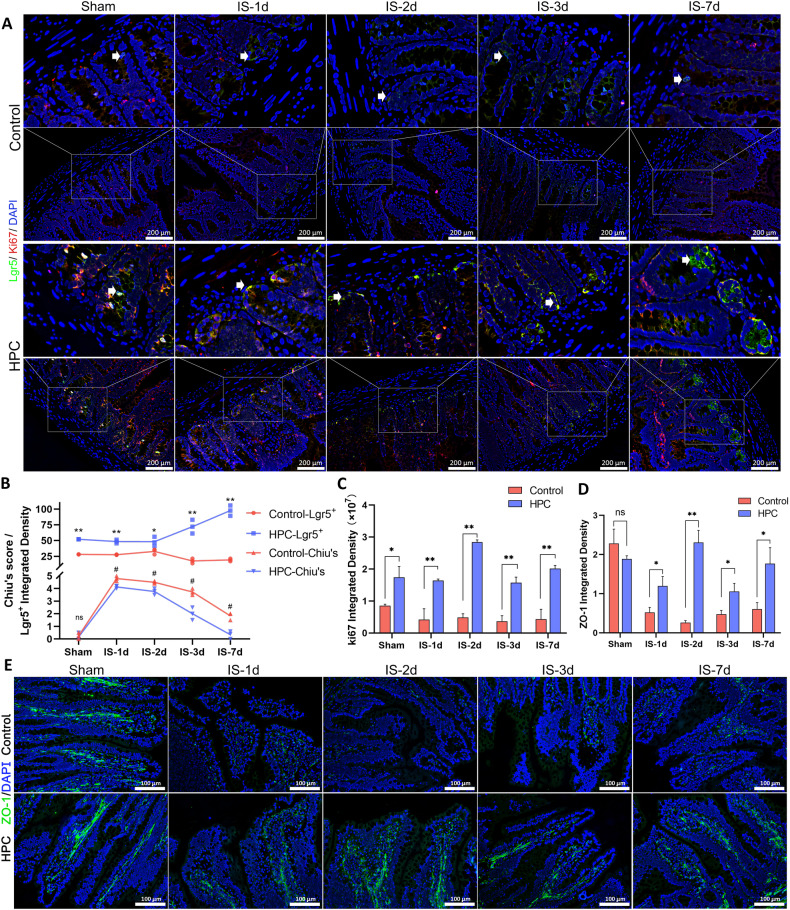
Distribution of stem cells in intestinal tissue. **A** Stem cells (Lgr5) and proliferative cells (Ki67) in the small intestine of rats in each group were labeled by immunofluorescence. **B** Chiu’s score and Lgr5 integral density analysis. **C** Ki67 integral density. **D** ZO-1 integral density. **E** ZO-1 was labeled using immunofluorescence. Values are listed as the mean ± SD. *p < 0.05, **p < 0.01 and ns, non-significant, compared with the control groups; ^#^*p* < 0.05 and ns, non-significant, compared with the control groups (*n* = 3).

Figure 2A depicts immunofluorescence labeling of intestinal stem cells (ISCs) expressing Lgr5 and proliferative cells marked with Ki67. The results indicated that the quantity of Lgr5+ cells at the base of the crypt in the HPC group was significantly more than that in the control group (Fig. 2B). In addition, there were more cells in the proliferative cycle in the HPC group (refer to Fig. 2C). The results above suggest that HPC treatment increased the number of intestinal stem cells in both the active and quiescent phases and promoted the proliferation of other cells, likely derived from the transition amplifying (TA) cells of ISC. We compared the trend of ISCs numbers with villus height/crypt depth based on H&E images and found that the number of ISCs in the HPC group was more and increased after the operation. Notably, this trend showed an inverse correlation with Chiu’s score, indicating that an increase in the number of ISCs was crucial for faster recovery from ischemic intestinal injury in the HPC group (Fig. 2B). Zonula occludens 1 (ZO-1) is an important component of tight junctions, and its expression level affects the function of the intestinal barrier. Our results showed that the expression of ZO-1 in the HPC groups was higher than that in the corresponding control group. In the HPC group, the intestinal barrier function recovered quickly (Fig. 2D, E).

### The expression of HIF-1α, PPARα, and CPT1A in intestine

**Fig. 3:**
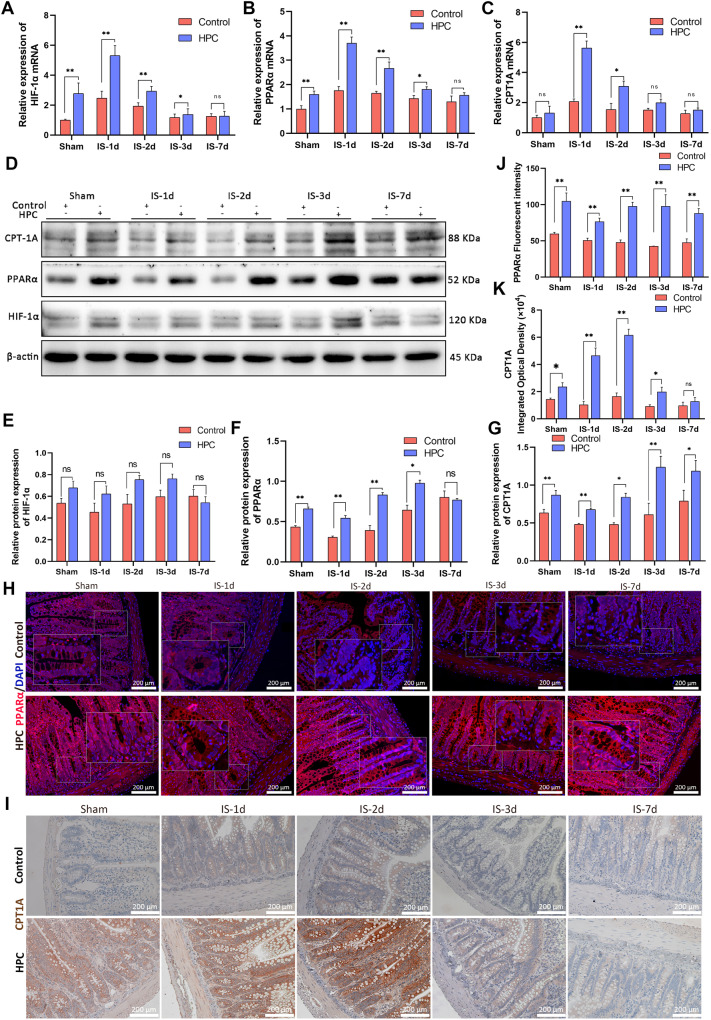
Activation of the HIF-1α/PPARα signaling pathway in HPC treated rats. **A**–**C** Contents of HIF-1α, PPARα and CPT1A mRNA in the intestinal tissues of rats in each group. **D**–**G** The expression of HIF-1α, PPARα and CPT1A in the intestinal tissues of rats were detected by western blot. **H**, **J** The distribution and expression of PPARα in the intestine of rats were detected by immunofluorescence. **I**, **K** The distribution and expression of CPT1A in the intestine of rats were detected by immunohistochemistry. Values are listed as the mean ± SD. Values are listed as the mean ± SD. *p < 0.05, **p < 0.01, and ns, non-significant, compared with the control groups (*n* = 3).

The results of real-time RT-PCR demonstrated that the mRNA levels of HIF-1α, PPARα, and CPT1A in the HPC groups were generally higher than those in the control group. On day 7, following ischemia, the mRNA levels of HIF-1α and PPARα returned to the levels observed in the control group (Fig. 3A–C). We utilized a western blot assay to determine protein expression and observed that following ischemia, PPARα and CPT1A levels were significantly higher in the HPC groups compared to the control groups. Moreover, PPARα levels returned to baseline on day 7 (Fig. 3D–G, Supplementary Information 1). Interestingly, despite the PCR results, we did not detect a significant increase in HIF-1α expression in the HPC groups. Based on existing literature, we surmise that the swift degradation of HIF-1α due to normal oxygen levels explains this experimental outcome [24]. In order to visually observe the distribution of PPARα and CPT1A proteins in intestinal tissues, we performed immunofluorescence and immunohistochemistry experiments (Fig. 3H, I). It was discovered that PPARα and CPT1A proteins increased in the HPC groups compared to the control groups, and CPT1A recovered to the control group level on day 7 (Fig. 3J, K).

### Activation of HIF-1α/ PPARα pathway in a 1% oxygen environment

**Fig. 4:**
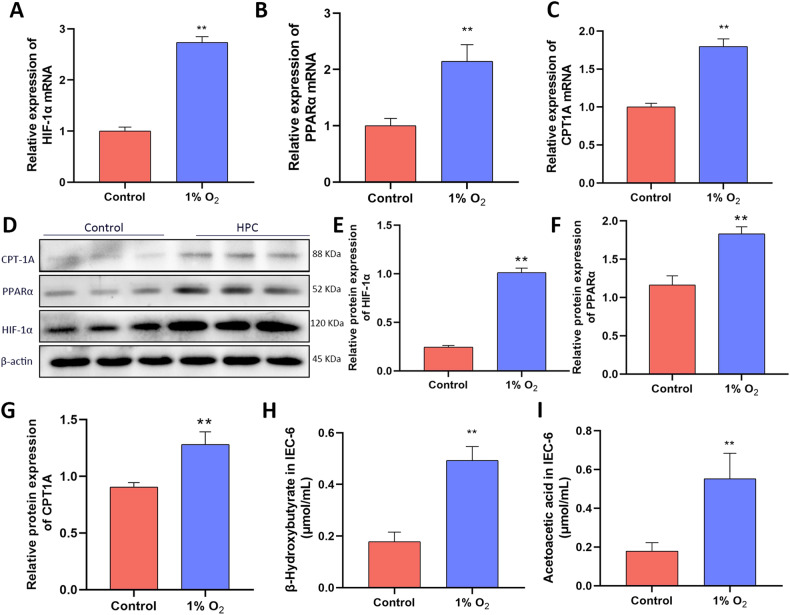
Activation of intracellular transcription and expression of HIF-1α, PPARα, and CPT1A in Low-oxygen environments. **A**–**C** HIF-1α, PPARα, and CPT1A mRNA levels in IEC-6 cells. **D** The expression of HIF-1α, PPARα and CPT1A proteins were detected by western blot. **E**–**G** Protein levels of HIF-1α, PPARα and CPT1A were analyzed based on western blot in IEC-6 cells. **H**, **I** β-hydroxybutyrate and acetoacetic acid levels were measured in IEC-6. Values are listed as the mean ± SD. Values are listed as the mean ± SD. *p < 0.05, **p < 0.01, and ns, non-significant, compared with the control groups (*n* = 3).

To investigate the impact of hypoxia on HIF-1α and its downstream genes, IEC-6 cells were exposed to 1% oxygen for 24 h, and the transcription and translation products of various genes were measured. The results showed that the mRNA levels of HIF-1α, PPARα, and CPT1A were significantly increased under hypoxic conditions (Fig. 4A–C). Western blot results showed that hypoxia significantly increased the protein levels of HIF-1α, PPARα, and CPT1A (Fig. 4D–G, Supplementary Information 1). In addition, β-hydroxybutyrate and acetoacetic acid also increased significantly (Fig. 4H, I). The above experimental suggesting that both transcription and translation of HIF-1α, PPARα, and CPT1A are activated in low-oxygen environment. Not only that, the FAO process is also activated.

### The transcription and translation levels of HIF-1α and its downstream genes after silencing the HIF-1α

**Fig. 5:**
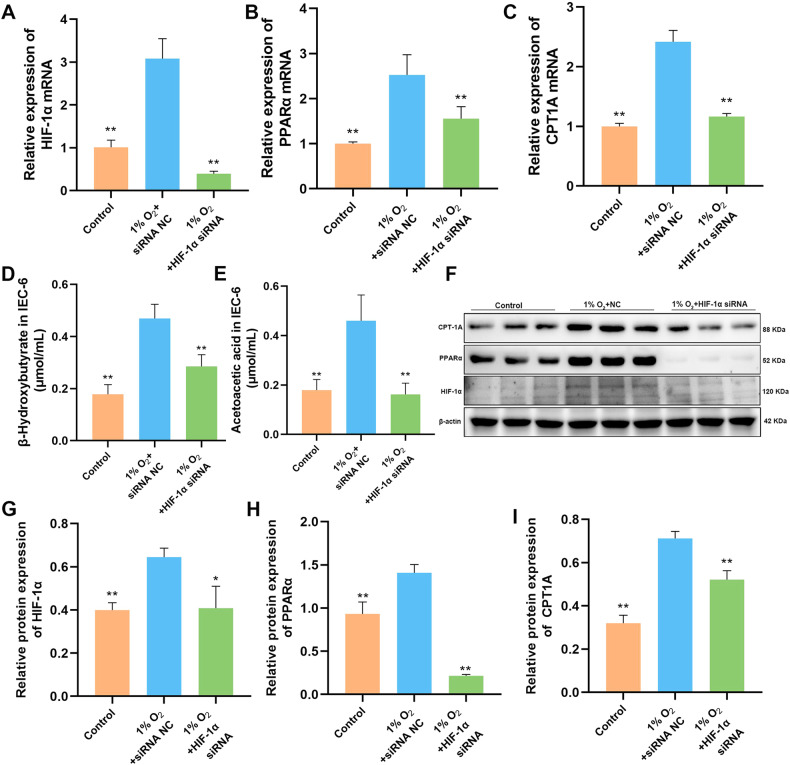
Inhibition of HIF-1α/PPARα signaling pathway after HIF-1α silencing in IEC-6. **A**–**C** After silencing HIF-1α, the mRNA levels of HIF-1α, PPARα, and CPT1A in IEC-6 cells decreased. **D**, **E** β-hydroxybutyrate and acetoacetic acid in IEC-6 cells were also significantly reduced after silencing HIF-1α. **F** The expression of HIF-1α, PPARα and CPT1A were detected by western blot. **G**–**I** After silencing HIF-1α, western blotting revealed a decrease in the protein levels of HIF-1α, PPARα, and CPT1A in IEC-6 cells. Values are listed as the mean ± SD. Values are listed as the mean ± SD. *p < 0.05, **p < 0.01, and ns, non-significant, compared with the NC group (*n* = 3).

We conducted experiments to silence the HIF-1α gene in order to gain a better understanding of the regulatory connection between HIF-1α and the downstream genes involved in FAO. After silencing the HIF-1α gene, the mRNA levels of HIF-1α, PPARα and CPT1A in IEC-6 cells were lower compared to the NC group (Fig. 5A–C). At the same time, the contents of β-hydroxybutyrate and acetoacetic acid in IEC-6 cells were also significantly reduced after silencing HIF-1α (Fig. 5D, E). Western blot test also showed that the expression of PPARα and CPT1A were inhibited after transfection with HIF-1α siRNA (Fig. 5F–I, Supplementary Information 1). Therefore, it can be shown that HIF-1α directly or indirectly regulates PPARα and CPT1A genes and interferes with FAO processes.

## Discussion

To investigate the impact of HPC on intestinal damage, a rat model was created utilizing a hypobaric chamber, followed by induction of intestinal ischemia through surgical means. We found that HPC treatment accelerated the restoration of the pathological structure following intestinal ischemic injury, increased the expression of the intestinal tight junction protein ZO-1, and decreased the entry of intestinal diamine oxidase into the systemic circulation, and this process was accompanied by the increase of the stem cell number. HPC increased the levels of β-hydroxybutyrate and acetoacetic acid, the oxidation products of fatty acids, as well as the transcription and translation products of HIF-1α and its downstream genes. HIF-1α interference experiment conducted in vitro confirmed the regulatory effect of HIF-1α on the PPARα and CPT1A genes. Thus, our data showed that HPC increases FAO and then encourages intestinal stem cells to take part in intestinal regeneration through activating HIF-1α and its downstream targets.

The viability of Lgr5^+^ cells is affected by several metabolites, and excessive cholic acid triggers ISCs dysfunction by inhibiting PPARα-mediated FAO [25]. Disruption of fatty acid β-oxidation inhibits the self-renewal of intestinal stem cells [26]. Increased ATP production through FAO was found to be the primary factor promoting the increase in Lgr5^+^ cells in the present study. The literature reports that CPT1A can directly mediate FAO and increase the levels of its byproducts, β-hydroxybutyric acid and acetoacetate, which aligns with our study [27]. We suggest that HPC is involved in the repair of intestinal injury by regulating FAO. The ISCs at the foot of the crypt receive enough energy from this pathway’s cascade. Highly mitochondrial stem cells feel the need to constantly divide and specialize into several cell types to replenish the damaged villus after intestinal injury [3, 28]. It has also been shown that fasting enhances ISCs function during aging by activating FAO [8]. Therefore, further investigation is warranted to explore the role of HPC in ISCs in aging rats. However, other studies have shown that activation of another member of the PPAR family, PPARδ, enhances the stemness and function of ISCs while also increasing the risk of adenoma. This raises the question of whether PPARα activation increases additional hazards in vivo, and further investigation is needed [8, 26].

In cell experiments, silencing the HIF-1α gene resulted in a decrease in the expression of PPARα and CPT1A. Surprisingly, there was no significant increase in HIF-1α protein, but there was a significant increase in HIF-1α mRNA in the HPC groups. Tracing the cause, we suspected that the HIF-1α protein was degraded after the rats returned from a hypoxic to a normoxic environment. The process involves the restoration of oxygen, during which prolyl and asparagine hydroxylase enzymes in the cytoplasm hydroxylate HIF-1α in an oxygen-dependent manner. HIF-1α then forms complexes with specific proteins for ubiquitination and subsequent degradation by the proteasome. The degradation rate of HIF-1α varies among different organs. The study showed that HIF-1α in the small intestine was degraded but remained higher than the control after 6 h. However, it returned to the baseline level after 24 h. Mice were exposed to hypoxia (6% O2) for 75 min. HIF-1α brain protein levels decreased to half of their initial levels within 15 min after the restoration of normal oxygen levels, and it was degraded after 60 min [24]. In this study, one possibility is that a 7-day HPC temporarily increases HIF-1α in the body and affects the PPARα/CPT1A pathway. However, the tissue has relatively low levels of HIF-1α content due to its rapid degradation.

We identified HIF-1α as a bridge connecting HPC to FAO. In addition, HIF-1α influences the gut through other pathways. Studies have shown that HIF-1α is a transcription factor that contributes to synergistic barrier protection. Activation of HIF-1α can regulate intestinal bacterial homeostasis and stabilize intestinal barrier function by increasing the production of antimicrobial peptides and upregulating P-glycoprotein and tight junction proteins. Intestinal HIF-1α exacerbates intestinal permeability, leading to bacterial translocation [29, 30].

As previously stated, our study indicates that HPC triggers HIF-1α/ PPARα-mediated FAO, thus facilitating intestinal restoration following ischemic intestinal injury, with an increase in ISCs acting as a pivotal point. This presents a potentially promising therapeutic approach for patients who suffer from intestinal disorders.

## Methods

### Animals

30 SPF-grade male SD rats weighing 250 g were randomly divided into two groups: A control group and an HPC group, with 15 rats in each group. Animal managers and experimentalists do not know the order of distribution; Researchers assessing, testing, or quantifying experimental results do not know the intervention. The Control group was fed normally. The HPC group was pretreated in a small animal hypobaric chamber for 7 days (2 h/day). Intestinal ischemia (a 45 min occlusion of the superior mesenteric artery) was performed on the eighth day. The rats were sacrificed under anesthesia on days 9, 10, 11, and 15, and the intestinal tissues were removed. The procedure for animal experiments is shown in Fig. 1A.

Animals were obtained from the Laboratory Animal Center at the Air Force Medical University and were approved by the Animal Ethics Committee of the Air Force Medical University before the experiment began. All experiments were performed in accordance with relevant named guidelines and regulations. The animals were euthanized by intraperitoneal injection of 200 mg/kg of sodium pentobarbital after the experiment. All animal experiments adhere to the ARRIVE guidelines.

### Cell culture and hypoxic treatment

IEC-6 cells were purchased from Shanghai Enzyme Research Biotechnology Co., LTD., and the cells were verified by STR. The cell lines were maintained at 37 °C and 5% CO2. Cell lines were cultured in 10% FBS, 1% P/S (Penicillin-Streptomycin), 0.1 u/ml insulin, DMEM, high glucose, and a medium containing pyruvate (ThermoFisher, 11995065). The cells were passaged when the cell density reached more than 80%. The HPC model was established at the hypoxic workstation with environmental parameters of 1% O_2_, 5% CO_2_, 94% N_2_, and a temperature of 37 °C for 24 h.

### Histopathological examination

The small intestine of the rats was rinsed with normal saline, and approximately 2 cm segments of the ileum were removed, fixed in 4% paraformaldehyde, embedded in paraffin, sectioned laterally, and stained with H&E (hematoxylin and eosin). The injury and recovery of intestinal tissue were observed under a microscope and evaluated according to Chiu’s scoring method [31]. Intestinal goblet cells were marked by AB/PAS staining kit (Solarbio Science & Technology, Beijing).

### Determination of FAO in vivo

The small intestine tissue was prepared into 10% homogenate, boiled in a boiling water bath for 10 min, mixed, and extracted for 1 min. The supernatant was taken at 3500 rpm for 10 min, and the ATP content was detected according to the kit instructions (Jiancheng Bioengineering Institutes, Nanjing). In addition, rat serum samples were used to detect the content of β-hydroxybutyrate and acetoacetic acid, and the operation procedures were as shown in the instructions (Solarbio Science & Technology, Beijing).

### Assessment of intestinal barrier dysfunction

Assessment of intestinal barrier dysfunctionIntestinal barrier dysfunction was assessed by measuring serum diamine oxidase (DAO) and intestinal Zonula occludens 1 protein (ZO-1) (1:100, Abcam, ab221547). Serum DAO activity detection kit (Solaibao Technology Co., Ltd., Beijing). All procedures were performed according to the manufacturer’s protocol (n = 3 per group).

### Immunohistochemistry

Small intestinal tissue was fixed with 4% paraformaldehyde, then embedded in paraffin and sectioned (about 3-4 μm thick). The paraffin sections were deparaffinized with xylene, dehydrated in an ethanol gradient, followed by hydration and antigen repair in a citrate high-temperature water bath, and blocked with BSA. Primary antibodies were incubated overnight at 4 °C ((CPT1A (1:1000, Abcam, ab234111), (LGR5 (1:400, Abcam, ab219107), Ki67(1:50, Abcam, ab279653)). The secondary antibodies were incubated at room temperature for 1 h, followed by microscopic observation.

### Quantitative real-time PCR assay

Total RNA was extracted from IEC-6 cells and the intestine using TransZolTM. cDNA was synthesized according to NovoScript®Plus All-in-one 1st Strand cDNA Synthesis SuperMix (gDNA Purge). Amplification was performed on the Roche LightCycler® 480 fluorescence quantitative system according to TB Green® Premix Ex Taq™ II (Tli RNaseH Plus) instructions. The relevant primer sequences are shown in Supplementary Table 1. β-actin was used to correct the mRNA levels, and the relative mRNA expression was calculated using the 2−^△△Ct^ method. Primer sequence is shown in supplementary Table 1.

### Western blotting assay

Cells and intestinal tissues were added to RIPA lysate (containing protease inhibitors) to extract protein samples, and the concentration of the samples was determined by the BCA method. Protein samples were loaded on SDS-PAGE gels (8-12%) and electrophoresed until the sample reached the bottom of the gel. The proteins were then transferred to a 0.45 um PVDF membrane (Millipore, USA) at a constant current of 300 mA. Next, sample membranes containing proteins were placed in a protein-free rapid blocking solution for 0.5 h at room temperature, followed by incubation with primary antibodies (HIF-1α (1:500, Novus, #NB100-105), PPAR alpha(1:1000, Abcam, ab215270), CPT1A (1:1000, Abcam, ab234111), β-actin(1:1000, Cell Signaling Technology, #3700 S) overnight at 4 °C. The next day, the membranes were incubated with HRP-labeled secondary antibodies (HRP-conjugated Goat Anti-Rabbit IgG (H + L) (1:10000, InCellGene, SA-10011)), or HRP-conjugated Goat Anti-mouse IgG (1:10000, Zhongshan Jinqiao Biotechnology, ZB-2305) for 1.5 h at room temperature. Finally, the ChemiScope 6000 Exp chemiluminescence imaging system and enhanced chemiluminescence were used to detect protein levels. β-actin was used to relatively correct the levels of proteins.

### Transfection experiment

Cells were inoculated with 5 × 10^3^ cells per milliliter. The transfection complex was prepared with riboFECT™ CP reagent, riboFECT CP buffer, and siRNA reserve solution at 50 nM and incubated at room temperature for 15 min. After the cells grew to 50% and fused, then they were added to the complete culture medium and mixed well. After 12 h of cell culture, the cells of each group were treated with hypoxia or normoxia.

### Statistical analyses

All statistical analyses were performed with Graphpad prism 8.0.2. Continuous variables were expressed as mean ± standard deviation (SD), there were three samples, and each sample repeated the experiment three times. The Shapiro-Wilk test was used to check the normality and homogeneity of variance of all data. For two-group comparisons, Student t-tests were used to determine differences between groups for normally distributed data. For multiple group comparisons, *p* values were analyzed using multiple t-tests. All tests were two-sided, and *p* <0.05 was considered statistically significant.

### Supplementary information

Supplementary Table

Original Data File

## Data Availability

The experimental data sets collected and/or analyzed during the current work are available upon reasonable request from the corresponding author. During the current investigation, no relevant resources were generated.
