# Supplementary material for: Hypoxic preconditioning accelerates the healing of ischemic intestinal injury by activating HIF-1α/PPARα pathway-mediated fatty acid oxidation
Source: Cell Death Discov. 2024 Apr 4;10:164. doi: 10.1038/s41420-024-01937-0 (PMC10994932; doi:10.1038/s41420-024-01937-0)
Supplement: Supplementary file 1 — Supplementary Table [file 41420_2024_1937_MOESM1_ESM.docx]

Supplementary Table 1. The primers sequences of mRNAs in the present work.

| Gene | Forward primer(5′-3′) | Reverse primer(5′-3′) |
| --- | --- | --- |
| HIF-1α | CCGCCACCACCACTGATGAATC | GTGAGTACCACTGTATGCTGATGCC |
| PPARα | TCAATGCCCTCGAACTGGAT | TGCTCTGCAGGTGGAGCTT |
| CPT-1A | CAGGAGAGTGCCAGGAGGTCATAG | TGCCGAAAGAGTCAAATGGGAAGG |
| β-actin | TGCTATGTTGCCCTAGACTTCG | GTTGGCATAGAGGTCTTTACGG |

F: forward primer; R: reverse primer.
