# Supplementary material for: Hypoxic preconditioning accelerates the healing of ischemic intestinal injury by activating HIF-1α/PPARα pathway-mediated fatty acid oxidation
Source: Cell Death Discov. 2024 Apr 4;10:164. doi: 10.1038/s41420-024-01937-0 (PMC10994932; doi:10.1038/s41420-024-01937-0)
Supplement: Supplementary file 2 — Original Data File [file 41420_2024_1937_MOESM2_ESM.docx]

**Uncropped blot**

fig.3D

β-actin 1



β-actin 2



β-actin 3



HIF-1α 1



HIF-1α 2



HIF-1α 3
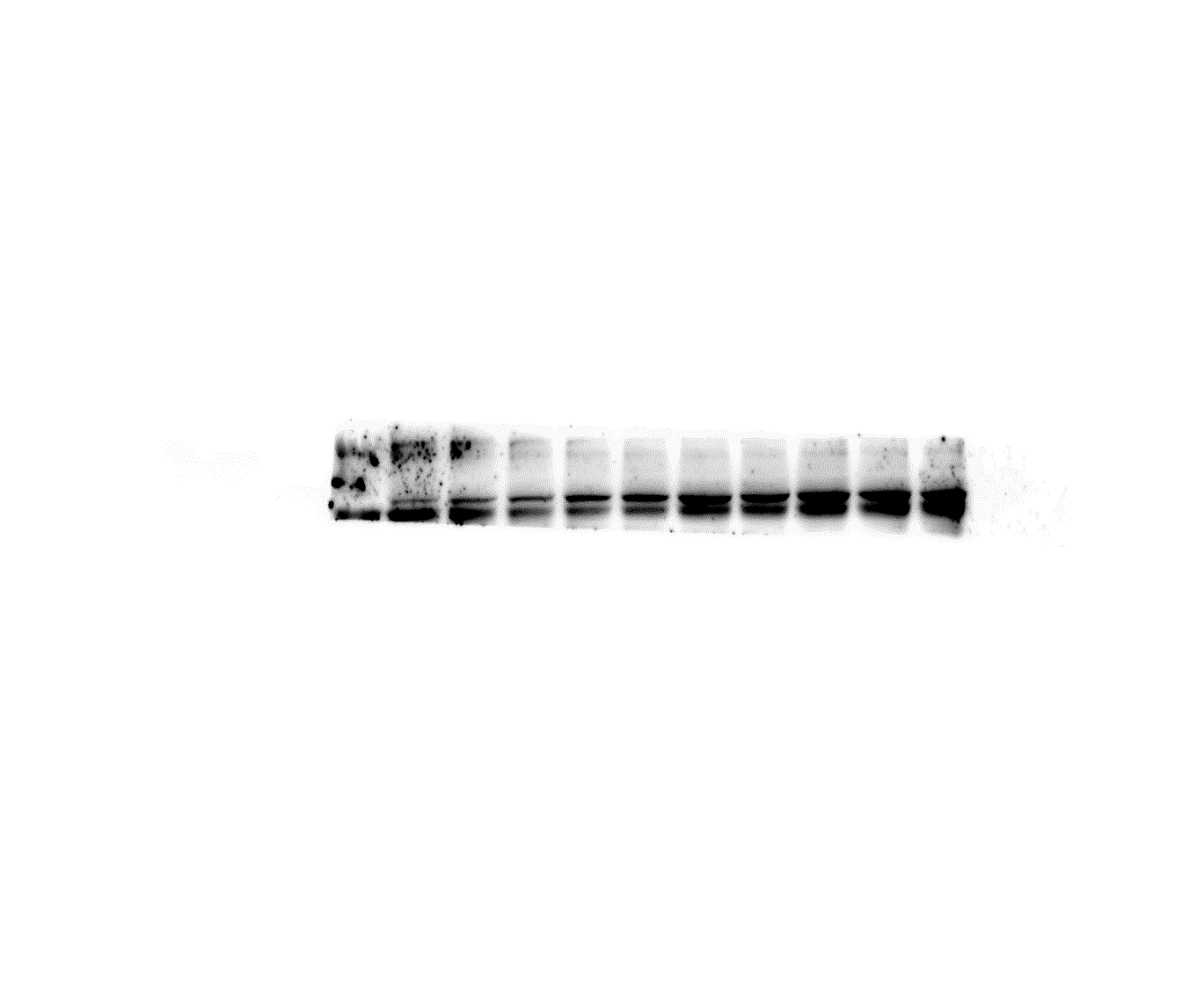


PPARα 1



PPARα 2



PPARα 3



CPT1A 1



CPT1A 2



CPT1A 3



fig.4D

β-actin 1



HIF-1α



PPARα



CPT1A



fig.5F

β-actin



HIF-1α
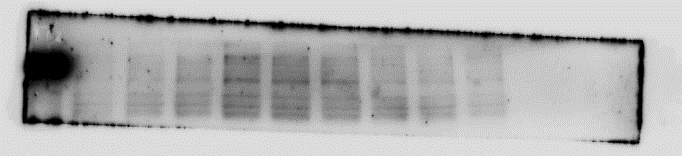


PPARα



CPT1A
